# Overview of influenza virus infections in Kenya: past, present and future

**DOI:** 10.11604/pamj.2013.14.138.2612

**Published:** 2013-04-08

**Authors:** Duncan Mwangangi Matheka, Jolynne Mokaya, Marybeth Maritim

**Affiliations:** 1 Department of Medical Physiology, University of Nairobi, Kenya; 2 Young Professionals Chronic Disease Network; 3 School of Nursing, University of Nairobi; 4 Department of Clinical Medicine and Therapeutics, University of Nairobi, Kenya

**Keywords:** Influenza, Kenya, surveillance network, Pandemic

## Abstract

The World Health Organization (WHO) estimates that acute lower respiratory infections account for 4 million deaths per year. The rates are even higher in developing countries. Influenza, a virus causing respiratory infections, has widely been studied in developed countries. However, there is paucity of data on its epidemiology, seasonality and burden in most developing countries. In the contrary, Kenya (a developing country) has an elaborate national epidemio-surveillance network for influenza, where a lot of data is generated on the epidemiology and seasonality of influenza in Kenya and the East African region. Several steps have been taken to control influenza in Kenya, including vaccination and surveillance programs. However, some challenges still exist. This article explores the pattern of influenza and existing interventions in Kenya, and highlights suggestions on what can be done to adequately control this virus in future.

## Introduction

Influenza is a respiratory infection caused by influenza viruses [1]. It spreads rapidly around the world during seasonal epidemics or pandemics and imposes a considerable economic burden [1]. Human influenza viruses are members of the orthomyxoviridae family. In humans, only influenza A and B viruses are of epidemiological interest [2].

The global burden of influenza on morbidity and mortality is high, with an estimated 1 million annual deaths worldwide [3]. In sub-Saharan Africa, little data on influenza exists, and poor disease surveillance makes the region ill-prepared to detect a new influenza strain or clusters of human cases that could be associated with an influenza pandemic [1, 4]. However, Kenya is among the few developing countries with elaborate national epidemio-surveillance networks for influenza. Besides, influenza still remains a major cause of hospitalizations and deaths every year in Kenya [5].

## Pattern of influenza virus in Kenya

Influenza occurs in distinct outbreaks of varying extents every year in Kenya. The epidemiologic pattern reflects the changing nature of the antigenic properties of influenza viruses, and their subsequent spread depends upon multiple factors including transmissibility of the virus and susceptibility of the population [2, 6, 7].

In Kenya, a tropical country, influenza is present throughout but with exacerbations at some times of the year [8, 9]. Influenza incidence tends to be highest in Kenya during a broad wave that mostly corresponds with the southern hemisphere winter, with peaks during wet months: March-April, October-November and cold month of July [9]. On the other hand, influenza in temperate countries comes in epidemics during the winter seasons.

Both influenza A and B are present in Kenya, with several strains of influenza already reported [9]. Percent type and sub-type of influenza in a study from 5th October 2006 to 8th November 2011 were: influenza B (31%), influenza A (H1N1) 2009 pandemic (28%), influenza A (H3N3) seasonal (24%), influenza A (H1N1) seasonal (10%) and unsubtyped (7%) [9].

## Previous influenza pandemics

Both seasonal and pandemic influenza infections have been reported in Kenya [10]. For instance, during the 1918-19 pandemic, the principal cause of deaths was bacterial pneumonia after influenza infections [11].

Avian pandemic influenza was reported in Kenya in 2006. It was also in other African countries especially Egypt which shares bird migratory pathways with Kenya [12]. This led to establishment of up to 26 surveillance sites for influenza in Kenya, surveying the different types of influenza including H1N1 (Swine Flu) and H1N5 (Avian Flu) [12]. An Avian Influenza National Strategic Emergency Preparedness and Response Plan was put in place, and has been used to guide influenza surveillance and response activities [12].

The recent 2009 pandemic influenza A (H1N1) virus was responsible for at least 20,000 laboratory-confirmed deaths globally [13]. In Kenya, the first-recognized case was identified on June 29, 2009, and by September 2009 a majority of influenza cases in the country were caused by H1N1 [14]. One Kenyan study carried out from July to November 2009, identified 690 patients with laboratory-confirmed pandemic (H1N1) 2009 [15]. Of these patients, 88 (13%) were hospitalized in 12 surveillance hospitals. Most of these hospitalizations (61, 69%) occurred during October and November, and the median patient age was 5.1 years. Several other studies have been conducted in Kenya to show the pattern of the H1N1 pandemic [16–18]. Data from these studies has being useful in influencing influenza-related policies.

In 2012, there was a steady decline of pandemic H1N1 influenza cases observed among the sentinel surveillance sites in Kenya. For instance, out of 745 samples collected between January 2012 and May 2012, only 92 were positive for influenza viruses; and influenza A constituted 86% of these samples. The rest of the samples were positive for influenza B [19]. Of the 80 samples positive for influenza A, 73 samples (91%) were seasonal H3N2, whereas the rest were influenza H1N1. Seasonal H3N2 has therefore almost currently replaced the pandemic virus across the country. There are still influenza B cases circulating concurrently with seasonal influenza H3N2. The seasonal influenza H3N2 remains the predominant influenza strain circulating in Kenya [19].

## Influenza co-infections and comorbidities

Many patients with influenza have more than one viral agent with co-infection frequencies reported as high as 20% [20]. There is also a clear link between influenza and pneumonia, with pneumococcal infection being a major cause of mortality during influenza epidemics [20].

A quarter of patients hospitalized with pandemic (H1N1) 2009 influenza in Kenya had an underlying medical condition, a lower proportion than reported in the United States (73%), Ireland (50%), and Chile (37%) [15]. The difference could be due to undetected chronic illnesses. Furthermore, in many health care facilities in Kenya, patients are not routinely screened for chronic diseases, thus data about chronic illness may not have been recorded on hospital charts [15].

Twenty percent of hospitalized pandemic (H1N1) 2009 patients with available HIV data were HIV positive. Although this percentage is higher than the national HIV prevalence (7%) [15], findings suggest that HIV positive patients could be at risk for severe pandemic (H1N1) 2009 influenza, supporting results of a South African study which reported that 53% of pandemic (H1N1) 2009 patients who died were HIV positive [15].

## Steps taken to control influenza in Kenya

Vaccination has been crucial in the control of influenza in Kenya, with a goal of protecting high-risk populations from severe outcomes. The US Advisory Committee on Immunization Practices recommends vaccination of children from six months to 12 years and adults aged 60 years and above against pneumonia to avoid deaths from influenza [21, 22].

In Kenya, the National Influenza Centre (NIC) in collaboration with the US Army Medical Research Unit - Kenya, Ministry of Public Health and Sanitation, Kenya Medical Research Institute (KEMRI) and WHO have been conducting surveillance in sentinel sites across the country [23]. This surveillance system was started at a time when the WHO had sounded an alarm on the possibility of pandemic influenza [24]. This system was to capture: influenza-like illness (ILI), Severe Acute Respiratory Illness (SARI) and Suspected Avian Influenza [23].

The above surveillance program has grown through the years since 2006, and is now a major component in the Kenyan Public Health infrastructure. In brief, nasopharyngeal swabs are collected from patients presenting with fever, cough or sore throat and the samples are transported to NIC for analysis [23]. Molecular and serologic influenza diagnostics, isolation of viruses, characterization of antigenic properties and mapping of genetic changes that are indicative of changes in virulence/antigenicity/drug resistance are carried out. The program is of benefit to other health facilities in the region as well [23].

In summary, Kenya has a working national epidemio-surveillance network comprising of both public and private professionals and other stakeholders. The response plan focuses on the following: epidemiological surveillance; information, education, communication and social mobilization; case management; laboratory and research; infection prevention and control; coordination and resource mobilization; human resource mobilization and additional emergency staff training [12].

## Challenges and recommendations in controlling influenza in Kenya

Besides the advances made in Kenya, a number of constraints still exist in the control of influenza. For instance, there are weak disease surveillance systems (in timeliness, completeness, human capacity). Therefore, it is prudent to strengthen such systems and also continue surveillance of influenza even after a pandemic has ended, so as to detect re-emerging and emerging diseases.

There is also inadequate support to both animal and human laboratories, hence inadequate laboratory capacity. This includes inadequate human resources. There are also inadequate emergency stocks of vaccines, anti-viral drugs, medical equipment, protective gear and other non-pharmaceuticals. Kenya should thus encourage more collaborations and get assistance from the international community in the following: Technical assistance (training, surveillance, diagnosis and case management), Mobilization of resources, International liaison and coordination, Harmonization of monitoring and evaluation indicators with international and regional levels, and strengthening the accredited and national laboratories (KEMRI and Central Veterinary Laboratory) to a level of regional/international influenza reference. Moreover, there are weak biosecurity systems at farms, veterinary laboratories and at entry points. There are also inadequate quarantine facilities for both animal and human at ports of entry. Kenya should thus strengthen such biosecurity systems, and ensure prevention of transmission between animals and humans.

There is low funding for research and thus inadequate prevalence studies in Kenya. Knowledge is limited regarding transmission of H1N1 in developing countries, where comorbidities (HIV/AIDS, malaria, and nutritional deficiencies) are more common than other locations. The effect of population density on H1N1 transmission patterns has not been studied too. Therefore, there is a role to promote more scientific research on influenza, as well as assess the disease magnitude and its impact on health and healthcare systems, as well as using the research outcomes to influence policy.

There is inappropriate legal framework to respond to the challenge. There are also unsynchronized communication systems. Kenya should therefore strengthen the legal framework, and encourage pandemic preparedness for human and animal diseases. It should also ensure sensitization of veterinary and human medical professionals and public through seminars, media, social networks, among other channels.

In Kenya as well as other developing countries, people are not well informed about the negative impact of influenza, its epidemiology, as well as how to prevent it. Kenya should thus promote public health interventions and educate people on the prevention of influenza. Early swabbing should also be promoted. Regarding prevention, Kenya should ensure better coverage of influenza vaccination programs. Efforts to vaccinate high-risk groups such as HIV-infected adults and those with underlying medical conditions should also be sustained. This includes vaccination for both influenza and pneumococcus. It should also ensure affordable antiviral treatment to those with confirmed influenza infection.

## Conclusion

Influenza still remains a threat to global health. Kenya, a developing country, has a relatively elaborate national epidemio-surveillance network for influenza. However, influenza-associated hospitalizations in Kenya are still high especially among children aged less than 5 years. Its co-infections and complications account for considerable morbidity and mortality. Multidisciplinary surveillance efforts are thus required in preventing influenza or its complications, and in ensuring pandemic preparedness in Kenya and other developing countries.
